# Healthcare, Responsibility and Golden Opportunities

**DOI:** 10.1007/s10677-021-10208-1

**Published:** 2021-06-14

**Authors:** Gabriel De Marco, Thomas Douglas, Julian Savulescu

**Affiliations:** 1 Uehiro Centre for Practical Ethics, University of Oxford, Oxford, UK; 2 Jesus College, University of Oxford, Oxford, UK

**Keywords:** Responsibility, Health, Healthcare, Golden opportunity

## Abstract

When it comes to determining how healthcare resources should be allocated, there are many factors that could—and perhaps should—be taken into account. One such factor is a patient’s responsibility for his or her illness, or for the behavior that caused it. Policies that take responsibility for the unhealthy lifestyle or its outcomes into account—responsibility-sensitive policies—have faced a series of criticisms. One holds that agents often fail to meet either the control or epistemic conditions on responsibility with regard to their unhealthy lifestyles or their outcomes. Another holds that even if patients sometimes are responsible for these items, we cannot know whether a *particular* patient is responsible for them. In this article, we propose a type of responsibility-sensitive policy that may be able to surmount these difficulties. Under this type of policy, patients are empowered to change to a healthier lifestyle by being given what we call a ‘Golden Opportunity’ to change. Such a policy would not only avoid concerns about patients’ fulfilment of conditions on responsibility for their lifestyles, it would also allow healthcare authorities to be justified in believing that a patient who does not change her lifestyle is responsible for the unhealthy lifestyle. We conclude with a discussion of avenues for further work, and place this policy in the broader context of the debate on responsibility for health.

## Introduction

When it comes to determining how healthcare resources should be allocated, there are many factors that could—and perhaps should—be taken into account. One such factor is a patient’s responsibility for his or her illness, or for the behavior that caused it. Should we take into account, for instance, the fact that a patient’s lung cancer is the foreseeable result of a lifetime of smoking, or that a patient’s heart disease is a foreseeable result of a lifetime of unhealthy eating habits? Call policies that take such considerations into account *Responsibility-Sensitive Healthcare Policies*, or *RSHP*s for short. On such policies, patients would, to some extent or other, be held accountable for their unhealthy lifestyles and/or their outcomes.

Some defend RSHPs^1^; while others reject them. One prominent line of attack gives reasons for thinking that patients sometimes, or perhaps always, lack responsibility for their health-related actions, or their outcomes, in virtue of lacking the requisite sort of control over them.^2^ Another worry is that patients might lack the sort of awareness of their actions, and their consequences, required to be responsible for these lifestyles and their negative health outcomes.^3^ Further, even if we suppose that *some* patients can and do have the requisite control and awareness over their actions and their outcomes, determining whether any *particular* patient has them is—some hold—either something that we cannot do, or involves doing things that we should not do (for example, being objectionably intrusive, or undermining the doctor-patient relationship).^4^

For the purposes of this paper, we will assume that human agents can sometimes freely and knowingly engage in health-risking lifestyles. Because of this, they can be responsible for having some of these lifestyles, and for some of the health outcomes of these lifestyles. However, we will also assume that, when a patient’s lifestyle is first discovered or suspected by the healthcare system, healthcare authorities are typically not in a position to know whether this particular patient is responsible for the illness that *may* be a result of her previous behavior, nor whether she met conditions on responsibility for the behavior itself. This may be because the healthcare authorities do not have enough evidence about the patient’s behavioral and medical history, and perhaps because gathering such evidence would be impractical or undesirable.

Even when making this second assumption, one might still be justified in taking responsibility into account when making healthcare decisions. This is because there are other ways of attaining knowledge about a patient’s responsibility for their unhealthy behavior. How might this be done? By implementing a policy of presenting patients with what we will call *Golden Opportunities* (GOs): opportunities to make a change to a healthier lifestyle at reasonable cost, and accompanied with appropriate support, should the patient choose to attempt the change. We elaborate on these features below, but some examples of possible GOs include offering, with appropriate support, e-cigarettes to smokers, methadone replacement therapy to heroin addicts, and nutritional or exercise programs to individuals at risk of type 2 diabetes or arterial disease (Savulescu 2017).

The reasons often given for uncertainty about the patient’s responsibility for her lifestyle stem from obstacles the agent may face in changing her lifestyle. Some patients may face obstacles to making a lifestyle change which reduce, or eliminate, their responsibility for the lifestyles in question. Given that we may not know, of a particular agent, whether, or to what extent, she faced such obstacles, we may not know whether she is responsible for her lifestyle. One of the aims of GOs is to help patients avoid as many of these obstacles as possible.

Once a patient is presented with a GO, we are in a significantly better position to determine the patient’s responsibility for at least some of her health-relevant choices—namely, whether to take or refuse the opportunity, and (at least for a period) whether to stick to the healthier lifestyle that she is offered. We also thereby remove some of the obstacles to implementing an RSHP. If the patient is responsible for the choice, and her choice was to decline the GO, we may then be justified in holding her responsible for that choice—and for whether she subsequently sticks to it—even though we are still not justified in holding her responsible for her behavior prior to the offer (Savulescu 2017). It is also plausible that, as others have argued, once the agent is responsible for declining the GO, “she cannot reasonably complain to being given lower priority on the waiting list” (Feiring 2008, p. 35) and “should be considered to have a weaker claim on resources than others who either did comply or never had a high-risk lifestyle in the first place” (Bærøe and Cappelen 2015, p. 838).

In order to determine whether GOs can indeed empower individuals, and reliably justify belief in the claim that the patient is responsible for some of her health-relevant choices and behaviors, we will need a fuller account of what a GO is than has been provided to date. In this paper, we develop the notion of a GO. Our main goal in doing so is to show how GOs might reliably help patients overcome obstacles to making the relevant change, and thus provide justification for the belief that such patients are responsible for some of their health-relevant choices and behaviors.

Before moving on, we wish to make some clarifications.

First, we should clarify what we mean by “responsibility.” This term is used in different ways in this debate. For instance, some speak of individual responsibility, others of personal responsibility, others of moral responsibility, and other still of “responsibility,” unqualified. We do not wish to take a stance on which of these notions is the relevant one in this debate, and simply stick to the general “responsibility.” What is important for our purposes is that responsibility has a control, and perhaps an epistemic, condition. The different sorts of responsibility often mentioned are often thought to have one, or both of these conditions.

However, there is also a different way of distinguishing between “types” of responsibility. Sometimes people contrast forward-looking, or prospective, responsibility with backward-looking responsibility. It seems to us that these terms are not used uniformly, though they are often intended to mark a difference similar to what we will call the obligation-sense and the accountability-sense of responsibility. Consider the claim that a doctor has a responsibility to his patients. Here, “responsibility” seems to refer to some sort of obligation or duty that a doctor has to his patients. Call this the *obligation-sense* of responsibility. Contrast this with the claim that John is responsible for failing to stop at the red light. Here, “responsibility” is not being used to refer to some obligation that John had; he certainly did not have an obligation to fail to stop at the red light. Here, we mean that John had the sort of control over his behavior, and awareness of (or capacity to be aware of) the relevant features, such that responding to his failure in a certain way is now appropriate. Call this the *accountability-sense* of responsibility.^5^ The sort of responsibility we are concerned with in this paper is this accountability-sense; GOs help to justify the belief that an agent is accountable for his choices and behavior, and RSHPs hold people accountable for their choices, behavior, and perhaps their outcomes.

Second, a patient offered a GO could be responsible for a variety of things: (1) the choice to accept/reject the offer, (2) the individual actions and omissions that constitute the lifestyle (new or old), (3) the lifestyle (that is, series of actions and omissions) itself, and (4) the health outcomes of the lifestyle. Our first assumption – that agents sometimes freely and knowingly engage in health-risking lifestyles – implies that individuals can be responsible for 1–3, and strongly suggests that they can be responsible for 4. For the majority of this paper, we will be concerned with patients’ responsibility for their choices after being presented with the GO (1), and for their ensuing lifestyles (2, 3), *not* their responsibility for the negative health outcomes of that behavior (4). When we speak of “the patient’s responsibility for her lifestyle,” or of her “responsibility for accepting or declining the GO” we intend to speak of the patient’s responsibility for one or more of 1–3, but not 4.

In taking this focus we do not mean to imply that agents are not, or cannot be, responsible for the outcomes of their lifestyles; nor do we mean to imply that a healthcare policy which held patients accountable for these outcomes would be unjustified. The main reasons for our focus on 1–3, as opposed to 4, are twofold (we elaborate on these further below). First, GOs can result in patients’ having the requisite control and knowledge over their choice and actions; yet having the requisite control over the outcomes of their lifestyles is a different matter, since there are various other factors involved which influence whether the lifestyle will result in a given outcome. Thus, GOs may not be as good at providing agents with the requisite control over the outcomes of their lifestyles. Second, given this point, and some further difficulty in acquiring evidence about the causal factors leading to an illness, we think the epistemic situation concerning responsibility for the outcomes of lifestyles will be a much more complicated matter.

Third, we wish to clarify that our main focus will be on GOs *as a component* of an RSHP, intended to solve some problems faced by such policies. In section 3, we suggest how such a policy can make use of features of GOs; yet we do not intend to develop a full version of such a policy. Further, we do not mean to imply that offering GOs would be desirable only insofar as it helps to justify such a policy; even if one thinks that RSHPs are always unjustified, one would still have reasons to implement a program of GOs, insofar as one wants to empower individuals to make changes in their lifestyles.^6^

Fourth, when we discuss RSHPs, we will be focusing on policies that make use of the knowledge provided by GOs, and are sensitive to responsibility for a lifestyle after the offer. Our discussion will be limited to considering the possibility that the responsibility of patients offered GOs might be one factor among others that determines the priority given to a patient, or it might modestly affect the proportion of treatment costs that the patient will be required to bear for health outcomes related to the lifestyle, or it might modestly increase the cost of premiums.^7^ When we speak of “holding the patient responsible,” we are referring to, and only to, these practices.^8^

We will not consider or defend a policy that recommends the absolute exclusion of some patients from the health care system or from public funding for healthcare. A policy of absolute exclusion from treatment or complete denial of funding, solely on the basis of responsibility, would face a host of objections which we do not address in this paper, nor are we convinced that they could be addressed adequately (Cohen and Benjamin 1991; Shiu 1993; Anderson 1999; Feiring 2008; Segall 2009).

Finally, a point about the sorts of health-related lifestyles we have in mind. RSHPs face what is sometimes called the *Universalization Objection*. Proponents of this objection argue, roughly, that holding people responsible for unhealthy lifestyles would require that we do so for many more lifestyles than are usually considered, and this would lead to absurd results.^9^ For instance, if we are to hold smokers responsible for smoking then, to be consistent, we should also hold responsible a physician who contracts Ebola while participating in a Doctors Without Borders project in an area where Ebola is prevalent; after all, both the smoker and the physician knowingly and willingly engaged in behavior that incurred risks to their health. But holding the physician responsible, say, by increasing the proportion of treatment costs that he is required to bear, would be absurd. The challenge is to delineate those health-related lifestyles which it would be appropriate to hold people responsible for in a way that avoids these implications.

For our purposes, we will remain neutral on how best to meet this challenge, focusing instead on paradigm cases of the sorts of lifestyles that are typically considered in this debate. However, one of us has addressed this objection in the past when introducing the original account of GOs (Savulescu 2017). The suggestion there was that one major factor will be whether the risks incurred by the lifestyle are unreasonable. The physician who contracts Ebola did not incur an unreasonable risk, whereas the smoker, at least once she has been presented with a GO, does incur an unreasonable risk by continuing to smoke. Determining whether a risk is reasonable or not will depend on a variety of factors. For example, the objective values of the costs and the benefits, the likelihood of the potential costs and benefits, whether there are means of minimizing likelihood of costs while maintaining benefits, etc. (Savulescu 2017).^10^

In the next section, we further develop the notion of a GO, offering a general framework for what a GO program would look like, and what it should aim to achieve if it is to form a component of an RSHP. In section 3, we make use of some of the discussion of GOs to suggest some desiderata for an RSHP policy that makes use of GOs. In section 4, we discuss areas for further development. We then offer some concluding thoughts on how our discussion fits into the broader context of the debate on RSHPs.

## Golden Opportunities

One of us has discussed the use of GOs in the past (Savulescu 2017, Brown and Savulescu 2019a, b; Davies and Savulescu 2019), and others have suggested similar notions, sometimes under the name of “forward-looking” or “prospective” responsibility policies (Feiring 2008; Bærøe and Cappelen 2015).^11^ In this section, we develop the idea, and add further conditions that we think an offer must meet to constitute a GO. Our goal will be in developing an account of GOs which remove obstacles to making a change in lifestyle such that 1) they empower individuals to make the change in lifestyle, and 2) they can justify the belief that the patient is responsible for their lifestyle, after the offer is made. The details of what constitutes a GO will depend on many features of the lifestyle under consideration, as well as the patient’s circumstances. Here, we simply intend to lay out some general features of GOs, and an RSHP that makes use of them, that helps to answer some of the challenges mentioned above.

As originally conceived, a Golden Opportunity is an offer of a lifestyle which:

1. has greater health-related value, and has greater, or at least not significantly less, non-health-related value for the patient^12^;
2. is realistically adoptable.

We elaborate on this below; but first, consider a rough sketch of a GO. Jim, a long-time smoker, has moderately severe obstructive airways disease from smoking. If he continues to smoke, he will likely require a lung transplant at some point in the future. Jim grew up in a poor, deprived, environment, and has been a smoker all of his life, just like his friends and family. He has found it difficult to quit on current smoking cessation programs. Jim’s doctors realize that if he continues to smoke, this will both reduce the chances that a transplant would succeed, and increase the chances that he will die prematurely without one. So, his doctors present Jim with a GO that would help him to quit smoking. They present him with various ways of making the change – e.g., switching to nicotine gum, nicotine patches, or a switch to e-cigarettes (E-cigs) – each of which would be accompanied with professional and peer support. Suppose Jim is considering making the switch to E-cigs, which contain nicotine, and deliver it through a similar process of inhalation, but also have vastly reduced health risks (Public Health England 2016; Royal College of General Practitioners 2017; Cancer Research UK 2019).^13^ With this switch, Jim could enjoy most, if not all, of the benefits that he currently derives from cigarette smoking. At the same time, switching to e-cigarettes would significantly lower the relevant health risks. Were Jim to reject the offer, or fail to adhere to the program after accepting it, we could be justified in giving him a lower priority for treatment related to the lifestyle, such as treatment for smoking-related obstructive airways disease, or in requiring him to pay a higher share of the costs of such treatment.

In order for an offer to reliably justify assignments of responsibility on the part of healthcare authorities, it will need to meet certain requirements. First, it is often thought that, in order to be responsible for an action, the patient needs to meet what is called the knowledge, or epistemic, condition: the patient must either be aware of the relevant features of her actions and their consequences, or, at least, capable of becoming aware of them.^14^ This leads to one of the worries mentioned in the introduction: some patients who engage in unhealthy lifestyles may not realize that they are incurring health risks, or they may not realize how significant the risks actually are. Further, some individuals may not be aware of ways of avoiding the risks. We think these worries are legitimate, and for the purposes of mitigating these worries, we suggest that the patient must be made aware of her options. She should be made aware of the potential consequences of both accepting and of declining the GO, both for her health, and for her entitlement to public healthcare and healthcare funding, and she should be made aware of the support that will be made available to her, were she to accept. For example, when presented with the GO, Jim should be made aware of the risks he is taking on, and will continue to take on, were he to continue smoking; both in terms to the potential health consequences, and the ways that the healthcare system might respond to his refusal. Further, he should be made aware of the various forms of support he is being offered, and the risks he would reduce by making the change. Of course, patients will have varying degrees of competence, and the nature of the communication with the patient may need to be sensitive to these differences in competence.^15^ Once made aware, the patient does not have, in virtue of the knowledge condition, a reasonable complaint against being held responsible.

Another concern arises from what is typically referred to as the control condition on responsibility. In order for an agent to be responsible for some action(s), she needs to have, or have had, the relevant sort of control over the relevant action(s). Because of this, it is important to understand the requirement that the offered lifestyle be ‘realistically adoptable’ such that satisfying the requirement reliably indicates that the patient had sufficient control over both 1) her decision concerning whether to accept or decline the offer, and 2) whether she in fact adopts the new lifestyle, should she choose to accept the offer. In order for a lifestyle change to be realistically adoptable in this sense, the offer will sometimes need to be accompanied with considerable support, where appropriate, in helping the patient make the lifestyle change (Davies and Savulescu 2019). Support could come in a variety of forms. In the case of Jim, for instance, e-cigarettes, their components, and the liquid (or cartridges) could be offered at a discounted price, and professional and peer support could be established to facilitate the transition (Savulescu 2017). If the offer involves a change to a healthier diet, support may include things like coupons for a price reduction for healthier foods. If the change involves exercising more, support might involve discounted gym memberships.^16^ The primary purpose of the support offered is to reduce or eliminate the obstacles that patients face when attempting to make a lifestyle change.

One such obstacle is the costs in making the lifestyle change. In their discussion, Bærøe and Cappelen suggest that any goods or services required to adopt the new lifestyle—such as e-cigs in the case of Jim—should be free of charge (Bærøe and Cappelen 2015, p. 839). This feature would help to defend against what they call the avoidability objection, which claims that, prima facie, it is fair to hold someone responsible for choosing a risk only if it could have reasonably been avoided. By making the support to the patient free of charge, the costs are no longer unreasonable, and the unhealthy lifestyle will not be unavoidable in virtue of the costs involved in making a change.

For the purposes of a general policy, we agree that the unhealthy lifestyle must be reasonably avoidable, and thus include it as a component of the realistic adoptability standard.^17^ However, the opportunity need not be free of charge in order to meet this requirement. What matters more, we suggest, is comparative cost. If the newer lifestyle incurs *fewer* costs than the risky lifestyle, then making the change in lifestyle will not be unreasonable in virtue of its being too costly.

Consider again the case of Jim. Using e-cigarettes already tends to be less expensive than smoking, and offering e-cigarettes, and e-cigarette products, at a discounted price further helps. With this policy, a patient could not justifiably claim that continuation of smoking is not reasonably avoidable because the alternative is too expensive. Of course, the lower the costs associated with the GO, the more reasonable it is to expect someone to take it (and thus to avoid the unhealthy lifestyle). This is so even if the costs of the GO are already lower than the costs of continuing the harmful behavior. The downsides of offering such a program free of cost is that the costs to the healthcare system of implementing GOs would be higher.

Whether the costs associated with the GO are unreasonable will depend, at least in part, on the patient’s economic conditions. One suggestion we offer here is an approach that takes into account a patient’s ability to pay for the costs associated with the change. On this approach, the discounts offered in the GO are sensitive to the patient’s financial status. An approach of this sort would help to mitigate some of the factors that are cause for concern over the patient’s responsibility for her lifestyle; and it would do so without making the healthcare system take on *all* of the costs for *all* patients who accept a GO.

Offering support at a reasonable cost should go some way towards reducing reasons for doubting whether a patient has the requisite control over whether she makes the desired change in lifestyle, yet it may not eliminate all of the barriers to making such a change. Socio-economic factors can present barriers to change that go beyond the price of the relevant products.^18^ For instance, some patients may be embedded in social situations where one does not get much social support for making the change, as with Jim, whose friends and family are all smokers. Or, if the change in lifestyle involves attending meetings, this may be more difficult for someone with children, who needs to arrange for childcare during the meetings. If possible, support should be offered to mitigate these worries.

Some individuals may also face a different sort of barrier to change. Consider the case of addiction.^19^ Addiction can significantly increase the difficulty of lifestyle change, and, at least in some cases, preclude responsibility for failing to make the change.^20^ Although we are hopeful that programs can be further developed to mitigate some of these concerns, and to make the changes in lifestyle easier, accommodating some of these may not be feasible. If, for a given lifestyle, there is no feasible offer we can provide that would resolve these problems—that is, if there is no feasible offer that can reliably provide the patient with the requisite control—then we do not have a GO available.

Yet it is important to distinguish between a patient’s having the control required for responsibility, and the degree of control the patient has above that level. Two patients may have significantly different degrees of control over a change in lifestyle *even if* they both have enough control to be responsible for the lifestyle (or the change). Some of the obstacles mentioned above may make it harder for the patient to switch to a healthier lifestyle, and thus may reduce the patient’s control; however, this need not imply that the patient does not have the control required for responsibility.^21^ Of course, the more support offered to the patient, the better the program is at empowering individuals to live healthier lives. But for a GO program to provide healthcare authorities with knowledge about patients’ responsibility, the support offered need not eliminate all obstacles for particular patients; merely reducing some of the obstacles may be sufficient in many cases. Thus, although it would be good to eliminate as many obstacles as possible, doing so is not necessary for an offer to be realistically adoptable.^22^

To sum up, then, a GO is an offer to change one’s lifestyle that is accompanied by appropriate support. Such an offer counts as a GO only if:

1. it has greater health-related value, and has greater, or at least not significantly less, non-health-related value for the patient;
2. it is realistically adoptable, where this implies that the existing lifestyle is reasonably avoidable;
3. the entity making the offer has ensured that the patient is well-informed about relevant matters (e.g., her options, potential health outcomes, the support offered, and the consequences of declining the offer); and

If, for a given patient with an unhealthy lifestyle, there is no feasible offer we can make that meets these conditions, we do not have a GO available.^23^ In the next section, we discuss how an RSHP that makes use of GOs might look.

## Responsibility-Sensitive Healthcare Policies

As suggested earlier, GOs will put healthcare authorities in a better position to know whether patients who continue to have unhealthy lifestyles after being presented with a GO are responsible for their lifestyles. However, we should stress that we are focusing on responsibility for lifestyles, rather than responsibility of the health *outcomes* of patients’ lifestyles.

The control a patient has over his or her decision to accept the offer, as well as the control the patient has over his or her adherence to it, is different than the control the patient has over his or her health status. GOs can help to remove obstacles to the patient’s deciding to make a lifestyle change, and to implementing that decision. However, there are many intervening factors between the agent’s decisions and actions, on the one hand, and the health outcomes of those actions, on the other; factors which are in many cases outside of the patient’s control, and can result in the patient’s having less control over the outcomes than she has over her decisions and actions. Moreover, most lifestyle-related illnesses can still occur in the absence of the behavior; non-smokers can get lung cancer, and healthy eaters can get type 2 diabetes and arterial disease. Consequently, although we may have good evidence to the effect that certain lifestyles are associated with certain illnesses, even when individuals exhibit both the lifestyle and the illness, we may still not be in a good enough position to know that a particular patient’s illness is due to the lifestyle. This uncertainty is something that every RSHP has to take into account, and one making use of GOs is no exception.

A further complicating factor derives from the fact that, *even if* we know that a particular patient’s illness is the result of an unhealthy lifestyle, we may still not know much about the patient’s responsibility for the illness, given one of our starting assumptions: that when a healthcare authority first learns of a patient’s lifestyle, they are not in a good position to know whether the patient was responsible for that lifestyle. Consider, for instance, a patient who has been smoking for the last 20 years, but only received (and refused) a GO five years go. Even if we know that his lung cancer is due to smoking, the GO helps us to know, at best, that he was responsible for the last five years of smoking. Consequently, although we may know that the lung cancer is a result of smoking, we may not be in a position to know that the lung cancer is due to smoking *for which the patient is responsible*. This is so especially in cases where the outcome is an illness related to a long-term lifestyle. The behavioral contribution to lifestyle-related illnesses often involves performing the same behaviors repeatedly, and over a long period of time (Brown and Savulescu 2019a). A clear picture of the causal contribution to the illness that distinguishes between behavior prior to the GO, and behavior after the GO, seems unachievable, given current knowledge. Although the change in lifestyle can improve the patient’s health in various respects, it may not be enough to prevent an illness resulting from behaviors the agent performed prior to accepting the GO. For these reasons, we suggest that when it comes to assessment of whether the patient has accepted and adhered to the GO, patients should not be assessed by health results, but rather by behavioral changes they adopt (Cappelen and Norheim 2005; Bærøe and Cappelen 2015, p. 839; Davies and Savulescu 2019).

In the previous section, we also made the point that agents who are responsible for their lifestyle might still differ in the degree of control they have over the changes to the lifestyle, and GOs may differ in how much they are able to reduce or eliminate obstacles to making the change. For some lifestyle changes, a GO may provide patients with sufficient control for responsibility, even though it might not provide them with a great amount of it. This will likely result in patients being responsible for their lifestyles to different degrees. One might then worry that a policy which did not take these differences in responsibility into account would be unfair; call this the *fairness concern*. There are at least two features of such a policy that could be varied in order to accommodate these differences in degrees of responsibility, and ease the fairness concern: (1) the standards used for what counts as adherence to the GO program, and (2) the extent to which failure to adhere may affect one’s priority or funding.

Consider, first, the standards used for determining adherence; should we, for instance, say that Jim has failed to adhere to his GO if, during a stressful period, he breaks down and smokes a few cigarettes? We think not, at least insofar as failure to adhere is taken as justification for holding Jim responsible. Missing a support-group meeting, or having a cigarette, should not be enough to constitute non-adherence. Such a standard would be unreasonable. The standard for adherence to a program should give some leeway; we should not require that patients adhere to it *perfectly*. How much leeway is given will depend on a variety of factors relevant to the difficulty in making the lifestyle change; e.g., how much of an imposition the program presents (for instance, if it requires attending meetings), whether the lifestyle change involves an addictive substance (and how addictive), and if so, whether the change involves a replacement (nicotine-replacement) or whether it requires abstinence (quitting alcohol), et cetera. Varying degrees of leeway can be used to accommodate varying degrees of difficulty in making the change in lifestyle. For those lifestyles which are harder to change, or for those patients who face more obstacles, adjustments in the leeway given will help to ease the fairness concern.

Another component of an RSHP using GOs that can ease the fairness concern is the way in which the healthcare system responds to responsibility. As we suggested in the introduction, we are considering a policy on which a patient’s responsibility for her lifestyle can affect either her priority for treatment of a lifestyle-related illness, the proportion of the costs of the treatment that she can be required to pay, in the form of a modest change in co-payment, or the cost of premiums. We have not mentioned, however, *how much* the patient’s priority, co-payment, or premium should be affected by her responsibility. The answer to this question, we think, can take into account the difficulty with making the change in lifestyle. Other things equal, the more difficult it is to make the change, the smaller the decrease in priority or subsidy, or the smaller the increase in premium, should be for those who fail to make the change. Further, for the version of the policy that requires the responsible patient to bear some of the costs of her treatment, we suggest the use of an ability to pay based approach. The costs the responsible patient is asked to bear should, in some way, take her ability to pay for them into account.

To sum up, if there is a GO available, and a patient declines it, or fails to adhere to it after initially accepting it, then one may be justified in giving the patient a lower priority for healthcare resources, or reduced funding. Yet the standard for adherence should be realistic about the difficulties involved in making the lifestyle change, and sensitive to these difficulties by providing the adequate amount of leeway. Further, the degree of the decrease in priority or subsidy ought to be sensitive to the difficulty in making the change. These last two features can help to ease the worry that differences in priority or subsidy offered to different patients would be unfair by failing to reflect differences in degree of control, and thus responsibility, for their lifestyles.

## Further Questions

There are some further questions which we do not address, and which would need to be addressed when fleshing out an RSHP making use of GOs. In this section, we mention some of these.

One interesting set of questions comes from the possibility of a patient receiving multiple GOs. Consider first the possibility that one patient declines two GOs for the same lifestyle, while another declines only one. Supposing that they are otherwise equal, should we give higher priority to the second patient, who declined only one GO? And if so, is it the absolute number of refusals that matters, or the number of refusals relative to the number of GOs that have been offered? For example, should we give higher priority to a smoker who was given, and declined, only one GO, than to a patient who was given and declined two? Is it relevant whether the difference in number of GOs received is due to the fact that one patient visits the doctor more often or gives more information about her past lifestyles?

These sorts of questions are complicated by the fact that a system that left GOs as a standing offer might be preferable. After all, one of the goals we want to achieve with healthcare policy is the improvement of health, and leaving the GO as a standing offer that a patient can take, even if she declined it in the past, would seem to be a better way of achieving this goal. Yet, if we leave GOs as standing offers that a patient can accept after initially declining them, we introduce difficult questions about what it is to decline a GO, and how, if at all, a policy should be sensitive to the length of time the GO was available but not taken. Suppose, for example, that a patient was offered a GO a year ago, which she originally declined. Further, suppose that it was left as a standing offer; one which she has not taken up. Should we say that, in some important sense, she has declined more than one offer?

Further, consider a case where a patient has multiple lifestyle factors for a single outcome. Suppose, for instance, that she has a bad diet *and* is a smoker; both being risk factors for heart disease. Now further suppose that this person accepts and adheres to a GO for obesity, but not for smoking. Were she to develop heart disease, should she get lower priority for treatment? Another question: should she get lower priority than someone who develops heart disease after a lifetime of smoking and refused GOs for smoking, but never had the other risk factor? Similar questions can be asked with regard to an RSHP which, rather than changing priorities, increased premiums. Questions like this are complicated by the fact that, although changing one’s lifestyle is possible, it can be difficult. Asking someone to change two aspects of her lifestyle (e.g., diet *and* smoking) at once may risk the possibility of making adherence to *both* offers, and perhaps *either*, too difficult.

Finally, consider the nature of the making of the offers themselves. We have operated with an ideal version of the policy: someone in the healthcare system makes the offer to the patient once the healthcare system becomes aware of the lifestyle. This gives us a relatively discrete event during which (a) the patient is made aware of potential consequences of continuing with their lifestyle or changing it, (b) obstacles to making this change are sufficiently mitigated to the point where the agent has the requisite control over the decision, and making the change, and (c) the healthcare system is aware that (a) and (b) are the case. Consequently, it gives us a case where the agent meets both conditions on responsibility for behavior *and* the healthcare system knows this. Would it be possible to achieve this result if patients were made the offer, and informed of the consequences, in a different way, say, through a concerted informational campaign?^24^ Communicating GOs in this way may result in more individuals becoming aware of GOs. However, it may reduce justification for believing that an individual met the epistemic condition on their behavior, and thus reduce justification for believing that they are responsible for their lifestyle.

## Conclusion

Consider, for a moment, a healthcare policy that implemented a system of GOs, yet did not hold people responsible for failing to accept or adhere to the offers. That is, consider a policy on which, once a healthcare professional discovers that a patient has a harmful lifestyle, the patient is informed of the consequences of this lifestyle, and offered significant support in helping to make a change in lifestyle, but on which declining to change lifestyle will not affect future priority or co-payments. We already have good reasons to implement such a policy. It would offer a way of helping people improve their health, and by doing so, it would result in better health outcomes for our society. Such a policy would do this while acknowledging the difficulties in making significant changes to lifestyles, and attempting to mitigate these difficulties. GOs don’t merely give us a way of recognizing when patients are responsible for their lifestyles; they enable people to take more control over their lives, and take responsibility for things that may have been too difficult without them. On many plausible understandings of it, this sort of program would increase people’s autonomy, with respect to their lifestyles, and the risks they want to take on.

These reasons in favor of implementing such programs remain, even when we add the responsibility-sensitive aspect of the healthcare policy. Moreover, implementing an RSHP may have some benefits over and above those associated with GOs alone. First, by changing the incentives patients have, it may motivate more patients to make a change to healthier lifestyles. Second, taking responsibility into account when making healthcare decisions may result in a fairer distribution of resources; particularly in a context where some resources may be scarce.

However, implementing an RSHP potentially raises various ethical concerns. We have shown how such a policy can avoid some reasons for concern. Assuming that we can be responsible for our health-related lifestyles, GOs can help patients avoid barriers to making changes in their lifestyles, barriers which threaten to undermine their responsibility for such behaviors. Given this fact, GOs can also provide healthcare authorities with evidence that the patients did, in fact, meet the relevant conditions on responsibility for their lifestyles. Further, in section 3, we addressed how an RSHP may address concerns about fairness when holding patients responsible.

There are, however, some further objections which we do not have room to address here, and which would not obviously be mitigated by GOs. For instance, we do not address the objection that an RSHP would be objectionably intrusive (Cohen and Benjamin 1991; Anderson 1999; Cappelen and Norheim 2005; Feiring 2008; Bærøe and Cappelen 2015; Véliz 2020), or that it would be impractical (Vathorst and Alvarez-Dardet 2000; Sharkey and Gillam 2010; Friesen 2018). We also do not address the concern that implementing an RSHP might damage the doctor-patient relationship (Glantz 2007; Ho 2008). These concerns apply, to some degree or other, to most RSHPs. Those whose objections to RSHPs are of these kinds are likely to be unmoved by the arguments offered in this article. However, those who object to RSHPs primarily on the basis of concerns about the patient’s lack of control or lack of awareness, or healthcare authorities’ lack of evidence for patient responsibility, may have good reasons to revise their positions if and when GOs are widely available.

## Footnotes

1 See (Moss and Siegler 1991; Cappelen and Norheim 2005; Feiring 2008; Buyx 2008; Thornton 2009; Bærøe and Cappelen 2015; Cavallero 2019).

2 For discussion, see (Vathorst and Alvarez-Dardet 2000; Resnik 2007; Ho 2008; Buyx 2008; Thornton 2009; Sharkey and Gillam 2010; Brown 2013; Bærøe and Cappelen 2015; Friesen 2018; Brown et al. 2019b, a; Davies and Savulescu 2019; Levy 2019a, 2019b; MacKay 2019; Brown and Savulescu 2019; Cavallero 2019)

3 For discussion, see (Resnik 2007; Sharkey and Gillam 2010; Davies and Savulescu 2019; Brown et al. 2019a; Brown and Savulescu 2019a).

4 For discussion, see (Cohen and Benjamin 1991; Vathorst and Alvarez-Dardet 2000; Cappelen and Norheim 2005; Daniels 2007; Ho 2008; Feiring 2008; Buyx 2008; Schmidt 2009; Sharkey and Gillam 2010; Brown 2013; Bærøe and Cappelen 2015; Friesen 2018; Brown et al. 2019b; Brown et al. 2019a; Davies and Savulescu 2019; Levy 2019a; Véliz 2020).

5 By making this distinction, we do not mean to suggest that these two senses are unrelated. For instance, it is quite plausible that the appropriate response to behavior that an agent is accountability-responsible for is in large part determined by the reasons and obligations the agent had in these circumstances.

6 We do not mean to imply here that the obligation is on individuals to improve their lifestyles, *rather* than on societies to adopt structures that are more conducive to healthy lifestyles (Cavallero 2019). It seems to us plausible that much of the obligation falls on society and that empowering individuals could be justified as one small part of a broader set of policies that would help to fulfil this obligation. Further, we do not mean to imply that a patient’s context will not play a (perhaps substantial) role in the explanation of their behavior, or resulting health outcome. However, this is consistent with the claim that such patients are, at least partly, responsible for their health-related lifestyles (and perhaps the outcomes of these lifestyles) (Schwan 2021).

7 Thus, we will not be considering policies that are sometimes discussed in these debates which would hold agents responsible by taxing goods or services related to the unhealthy lifestyle.

8 One problem that RSHPs sometimes face is that by modifying priority, or increasing proportion of treatment costs a patient should bear in light of the patient’s responsibility, one is only holding responsible those patients who ended up with the negative health outcome, rather than all of those who engaged in the unhealthy lifestyle. We will not address this issue here. Yet, it is important to point out that this problem does not seem to apply to RSHPs which modify the cost of premiums in virtue of a patient’s responsibility for their unhealthy lifestyle.

9 See (Shiu 1993; Cappelen and Norheim 2005; Buyx 2008; Sharkey and Gillam 2010; Friesen 2018).

10 For a possible alternative response, see (Segall 2009). On that view, it is permissible, in some cases, to treat agents differently with regard to negative health outcomes which it would have been reasonable for society to expect them to avoid.

11 Moss and Siegler also suggest that alcoholics might be responsible for needing a new liver, not in virtue of being responsible for drinking, but perhaps in virtue of being responsible for not seeking treatment (Moss and Siegler 1991). Vansteenkiste and colleagues suggest a policy on which patients with unhealthy lifestyles are offered “second chances,” but this policy is not an RSHP, in our sense (Vansteenkiste et al. 2014).

12 By “value” we mean “objective prudential value”, i.e. “contribution to well-being”. This is based on previous formulations, which can be found in (Savulescu 2017; Davies and Savulescu 2019). Previous formulations stated 1 as a disjunction, and the second disjunct allowed for greater non-health-related value, but similar health-related value as the existing lifestyle. We omit this second disjunct since it is doubtful that the health system should be in the business of promoting lifestyles with no greater health-related value than the patient’s current lifestyle. Further, we should clarify that the health-related value is independent of the healthcare system’s holding patients responsible in virtue of their responses to the GO.

13 Some might worry about the recent outbreak in the US of e-cigarette, or vaping, product use-associated lung injury (EVALI). Current evidence suggests EVALIs are strongly linked with vaping tetrahydrocannabinol (THC), not nicotine; and in particular, with the vitamin E acetate sometimes used to dilute the THC product (Fairchild et al. 2019; Centers for Disease Control and Prevention 2020; U.S. Food and Drug Administration 2020). Though there is a need for further research, these results suggest that concerns regarding EVALIs are not a significant concern with e-cigarette products intended as a nicotine replacement, as opposed to a delivery method for THC.

14 The precise nature of the knowledge condition is a matter of dispute, and as some have suggested, it may be much more related to the control condition than is typically thought (Mele 2010; Levy 2011, chap. 5).

15 If a patient is *incapable* of understanding the choice they are confronted with, then it would seem that a GO is not available for her.

16 We do not mean to suggest that offering these examples of support will be *sufficient*. Determining how much support is appropriate will be a complicated matter, and will be different for different lifestyle changes.

17 We agree with Bærøe and Cappelen that, in some cases, a person may be responsible for her lifestyle (and its outcomes) even if the risks are not reasonably avoidable (Bærøe and Cappelen 2015, p. 838). However, it is not clear how common these cases are, nor is it clear how we could reliably know that a patient is in one of these cases. This is why we take the standard of reasonable avoidability seriously (as do Bærøe and Cappelen), and incorporate it at the more general level of policy.

18 For discussion of such factors in the context of healthcare and responsibility, see (Vathorst and Alvarez-Dardet 2000; Cappelen and Norheim 2005; Resnik 2007; Daniels 2007; Ho 2008; Feiring 2008; Buyx 2008; Thornton 2009; Sharkey and Gillam 2010; Brown 2013; Bærøe and Cappelen 2015; Brown et al. 2019b, a; Davies and Savulescu 2019; Levy 2019a, 2019b; MacKay 2019; Cavallero 2019)

19 For discussion of addiction in the context of healthcare and responsibility, see (Vathorst and Alvarez-Dardet 2000; Resnik 2007; Buyx 2008; Thornton 2009; Sharkey and Gillam 2010; Brown 2013; Bærøe and Cappelen 2015; Friesen 2018; Levy 2019a, 2019b; Brown and Savulescu 2019a; Cavallero 2019; Véliz 2020)

20 One might think that, even if addictive desires do make it such that the agent does not have the requisite control over her decisions or actions that is from the desire, she may still be indirectly, or derivatively, responsible for such actions. This would be so if the agent’s responsibility for such actions can be traced back to some prior action(s) that she performed which led to the addiction, an action (or actions) over which she *did* have the relevant control and awareness. Although we find a general tracing strategy attractive, this move is not available to us here. Since we are assuming that healthcare authorities, when they first discover the unhealthy lifestyle (and addiction), are not in a position to know whether the patient was responsible for prior behavior related to the lifestyle, they are also not in a position to know whether there is such an action(s) that the addictive desire can be traced back to.

21 For a discussion of the relationship between difficulty and degrees of *moral* responsibility, see (Nelkin 2016).

22 One further type of factor sometimes mentioned in this context is genetic factors, though this is often mentioned in passing (Vathorst and Alvarez-Dardet 2000; Ho 2008; Buyx 2008; Thornton 2009; Sharkey and Gillam 2010; Bærøe and Cappelen 2015; Friesen 2018; Davies and Savulescu 2019; MacKay 2019; Cavallero 2019; Véliz 2020). In this context, genes are thought to threaten responsibility for health outcomes (e.g., a genetic predisposition for obesity), and responsibility for behavior (e.g., a genetic predisposition to become addicted to alcohol). As will become more apparent in the next section, we are concerned with responsibility for the latter, not the former. Insofar as genes are related to responsibility for behavior, they will be relevant via other things we do consider, i.e., the strength and/or addictiveness of desires.

23 We have not, in this paper, elaborated on 1 above. For a short paper focusing on that component, see (Savulescu 2017).

24 Of course, the support offered would remain the same; the difference here is in the means of communication.
